# Remodeling of the mitochondrial quality control network: natural products intervening in diabetic retinopathy

**DOI:** 10.3389/fphar.2026.1851846

**Published:** 2026-06-23

**Authors:** Tingru Chen, Ruolan Wu, Xinyi Liu, Yutong Jiang, Qun Huang, Ninghui Wan

**Affiliations:** 1 Chengdu Women’s and Children’s Central Hospital, School of Medicine, University of Electronic Science and Technology of China, Chengdu, China; 2 Chengdu University of Traditional Chinese Medicine, Chengdu, China; 3 Hospital of Chengdu University of Traditional Chinese Medicine, Chengdu, China; 4 Traditional Chinese Medicine Hospital of Meishan, Meishan, China

**Keywords:** diabetic retinopathy, mitochondrial biogenesis, mitochondrial dynamics, mitophagy, natural products

## Abstract

Diabetic retinopathy (DR) remains a leading cause of vision loss among working-age adults. Its pathogenesis is increasingly understood as the progressive dysregulation of the mitochondrial quality control (MQC) network, which encompasses mitochondrial dynamics, mitophagy, mitochondrial biogenesis, mitochondria-associated endoplasmic reticulum membranes (MAMs), and intercellular mitochondrial transfer. Under sustained hyperglycemia, this network shifts from compensatory imbalance to irreversible collapse, driving mitochondrial dysfunction, oxidative stress, inflammatory activation, and retinal neurovascular unit (NVU) injury, thus promoting progression from non-proliferative to proliferative DR. Because of their multitarget properties, natural products (NPs) can restore fusion-fission balance, modulate mitophagy in a stage-dependent manner, and promote mitochondrial biogenesis, thereby remodeling the MQC network. However, their clinical translation is constrained by low bioavailability, poor penetration of the blood-retinal barrier (BRB), and potential dose-dependent toxicity. Mitochondria-targeted nano-delivery systems, including liposomes, exosomes, and mitochondrial-derived vesicles, may improve retinal accumulation and mitochondrial targeting. Future studies should refine stage-specific mechanistic understanding, strengthen safety evaluation and structural optimization, and integrate single-cell omics with artificial intelligence to accelerate translation and enable early MQC-targeted intervention, with the potential to delay or even reverse the progression of DR. Critically, MQC-directed NP therapy should not be interpreted as uniformly pro-mitophagy, anti-fission, or pro-biogenesis; the therapeutic benefit depends on disease stage, cell type, autophagic flux integrity, target engagement, and retinal pharmacokinetic/pharmacodynamic exposure.

## 1 Introduction

Diabetic retinopathy (DR) is a microvascular complication of diabetes and the main cause of blindness in working-age adults worldwide (Qiu et al., 2024). With the increasing prevalence of diabetes, the incidence of DR continues to rise. Epidemiological data show that the global prevalence of DR is about 22.27%, and it is expected that by 2045, the number of affected individuals will reach 160.5 million (Wang Z. et al., 2024). DR progresses from non-proliferative diabetic retinopathy (NPDR) to proliferative diabetic retinopathy (PDR). Although NPDR usually does not cause obvious visual impairment, there are microvascular abnormalities, including vascular leakage and retinal hemorrhage (Pan et al., 2025). PDR is mainly driven by retinal ischemia, which is characterized by neovascularization, vitreous hemorrhage and tractional retinal detachment. These changes are the main cause of severe vision loss in patients with diabetes (Li et al., 2020). Therefore, early intervention in diabetic retinopathy is crucial to delay the progression of the disease and prevent irreversible vision loss.

At present, the main treatments for DR include intravitreal injections of anti-vascular endothelial growth factor (VEGF) drugs and panretinal photocoagulation (Hao et al., 2024). Anti-VEGF drugs inhibit vascular leakage and pathological neovascular formation by inhibiting key pathogenic mediators. However, their therapeutic effect is usually not durable and requires repeated intravitreal administrations. In addition, they cannot repair the retinal ischemic lesions that have been formed, and the treatment effect is ineffective for some patients. Panretinal photocoagulation reduces retinal oxygen consumption and reduces the production of pathological factors by ablation of the peripheral retina, thus producing a relatively lasting therapeutic effect. However, this therapy itself is destructive. It may have an irreversible impact on retinal function, leading to peripheral visual field defects and night vision disorders, and inducing or aggravating macular edema. In general, these methods mainly stabilize late vascular lesions and prevent blindness, but they cannot reverse early neurodegenerative changes. Therefore, exploring new therapies to protect neurovascular unit (NVU) in the early stages and delay or reverse neurodegenerative processes has become an urgent research focus.

**FIGURE 1:**
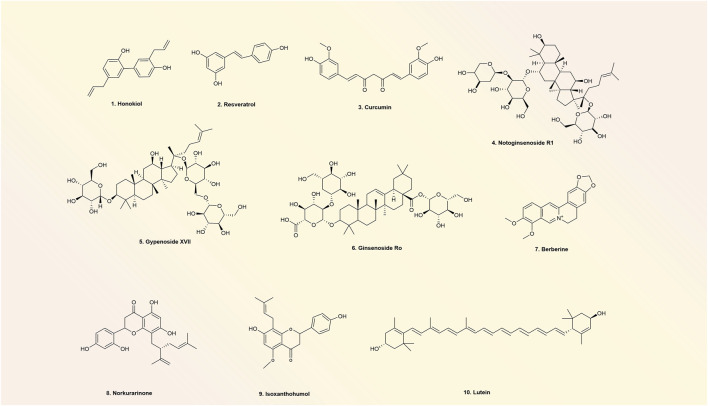
Structural frameworks of natural products involved in remodeling the mitochondrial quality control network in diabetic retinopathy. By summarizing the polyphenols, saponins, flavonoids, and alkaloids included in this review, we establish a comprehensive research framework showing how these NPs protect against diabetic retinopathy by orchestrating core mitochondrial quality control processes, including mitochondrial dynamics, mitophagy, and mitochondrial biogenesis.

In recent years, our understanding of the pathogenesis of DR has undergone a profound paradigm shift, from a focus on isolated mitochondrial dysfunction and apoptosis to a systematic understanding of the mitochondrial quality control (MQC) network. This network is a finely regulated dynamic system that maintains mitochondrial morphology, quantity, and quality through core processes such as mitochondrial dynamics, mitophagy, and mitochondrial biogenesis (Song et al., 2024). These processes are coordinated by interactions between mitochondria and the endoplasmic reticulum as well as by intercellular mitochondrial transfer. Studies indicate that the collapse of the MQC network is a key upstream event in the pathogenesis of DR, leading to mitochondrial dysfunction, redox imbalance, and inflammatory responses. This, in turn, disrupts NVU homeostasis, causing retinal microvascular injury and neurodegenerative changes and thus driving the progression of DR. A particularly challenging aspect of this process is metabolic memory. In this state, hyperglycemia-induced epigenetic modifications, including DNA methylation and histone deacetylation at the peroxisome proliferator-activated receptor gamma coactivator 1-alpha (PGC-1α) promoter, persistently suppress PGC-1α expression even after glycemic normalization. This prevents mitochondrial functional recovery and helps explain why DR can continue to progress despite strict glycemic control. Within this framework, natural products (NPs) may offer advantages over single-target synthetic small molecules. Their structural diversity enables simultaneous intervention at multiple nodes of the MQC network. Various NPs help maintain MQC network stability by regulating mitochondrial dynamics, fine-tuning mitophagy in a stage-dependent manner, and promoting mitochondrial biogenesis. However, research on NPs for DR still faces numerous bottlenecks. Most studies remain limited to *in vitro* systems and animal models. Critical breakthroughs are urgently needed in the precise delineation of *in vivo* mechanisms, the selection and optimization of active compounds, the improvement of pharmacokinetic properties, and the validation of safety and efficacy during clinical translation (Figure 1).

Nevertheless, the current evidence base should be interpreted with caution. Most mechanistic data are derived from high-glucose cell culture, STZ or db/db rodent models, and zebrafish models. These systems do not fully reproduce human NPDR and PDR heterogeneity, chronic metabolic memory, comorbid vascular risk, or long disease duration. Therefore, conclusions regarding the efficacy of NPs should be explicitly stratified by evidence level, model system, disease stage, and retinal cell context.

## 2 The mitochondrial quality control network in diabetic retinopathy: pathological mechanisms and regulatory hubs

**FIGURE 2:**
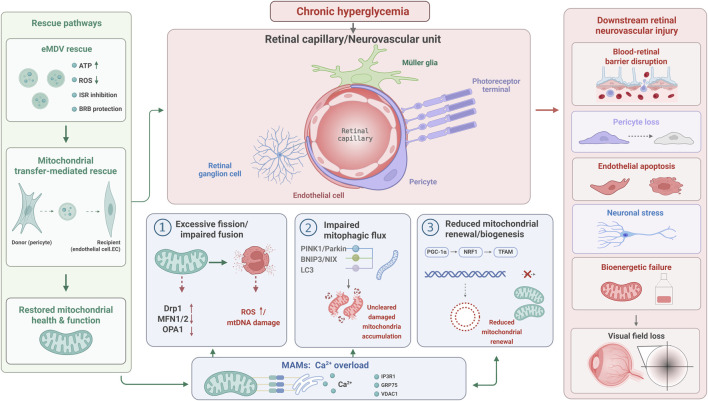
Pathological mechanisms and therapeutic opportunities associated with mitochondrial quality control collapse in diabetic retinopathy. The diagram illustrates how persistent diabetic stressors, including hyperglycemia, AGEs, and ROS drive the global collapse of mitochondrial quality control within the retinal NVU. This collapse manifests through five interconnected dysfunctions: mitochondrial dynamics imbalance covering fusion and fission defects, mitophagy failure leading to damaged mitochondria accumulation, suppressed biogenesis resulting in energy metabolism collapse, MAMs dysfunction characterized by ER-mitochondria miscommunication, and defective intercellular mitochondrial transfer. These molecular defects trigger cellular damage across endothelial cells, pericytes, retinal ganglion cells, Müller cells, and retinal pigment epithelial cells, thereby driving progression from early DR features, such as pericyte loss and BRB breakdown, to advanced DR and PDR features, including neovascularization, neurodegeneration, and vision loss.

Dysregulation of the MQC network is central to the development and progression of DR. Temporal and spatial imbalances, together with aberrant interactions among mitochondrial dynamics, mitophagy, and mitochondrial biogenesis, collectively drive the transition from mitochondrial dysfunction to irreversible retinal damage (Figure 2). This integrated network provides a theoretical foundation for identifying intervention targets for NPs.

### 2.1 Dysregulation of mitochondrial dynamics in diabetic retinopathy: mechanistic insights

Mitochondria are highly dynamic organelles that continuously undergo fusion and fission, altering their shape, size, and position. These processes, collectively termed mitochondrial dynamics, maintain mitochondrial homeostasis (Yan et al., 2024). Fusion can promote the exchange of mitochondrial contents and help to dilute the damaged components while division supports the redistribution of mitochondria, respiratory adaptation and separation of damaged mitochondrial fragments for autophagy degradation (Jackson and Theiss, 2020; Yan et al., 2024). These processes are mainly regulated by small GTPases, including dynamin-related protein 1 (Drp1) that mediates fission, and mitofusin 1/2 (MFN1/2) and optic atrophy 1 (OPA1) that regulate the fusion of mitochondrial outer membrane and inner membrane respectively (Cai et al., 2022). However, it is important not to regard mitochondrial division as an essential pathological process. Basal transient fission is necessary to separate damaged mitochondrial fragments and allow subsequent mitophagy; therefore, complete inhibition of mitochondrial division may damage MQC rather than restore it (Zhang J. R. et al., 2026). When Drp1-dependent division is excessive, continuous or decoupled from subsequent mitophagy, pathological phenomena will occur, leading to mitochondrial fragmentation, reactive oxygen species (ROS) amplification, cytochrome c release and apoptosis.

In DR, chronic hyperglycemia can shift mitochondrial dynamics in the direction of excessive division and fusion damage. In retinal microvascular endothelial cells, Drp1 is overactivated, while MFN2 expression is inhibited. This imbalance will promote mitochondrial fragmentation, reduce respiratory efficiency and ATP production, and increase ROS production (Kowluru and Alka, 2023). Increased ROS will further damage mtDNA and aggravate mitochondrial dysfunction and oxidative stress (Mishra and Kowluru, 2015). At the same time, Downregulation and proteolytic cleavage of OPA1 will change the integrity of the cristae and promote cytochrome c release, which leads to endothelial cell apoptosis, increased vascular permeability and thickening of the capillary basement membrane. Other retinal cells also have similar disorders, although their upstream triggers are different. In retinal pericytes, high glucose increases the level of cAMP and activates the exchange protein directly activated by cAMP 1 (Epac1). Epac1 promotes Drp1 phosphorylation and excessive division of mitochondria, impairing mitochondrial integrity and activating the intrinsic apoptosis pathway (Yang et al., 2023). Pericyte apoptosis will weaken capillary support, accelerate the formation of microaneurysms, and increase vascular leakage. In retinal ganglion cells, high glucose may upregulate miR-122-5p and inhibit DJ-1, thus promoting mitochondrial fragmentation, ROS accumulation and ganglion cell apoptosis (Peng et al., 2025). These changes will lead to degeneration of the optic nerve and visual field defects.

In general, aberrant mitochondrial dynamics is an early upstream event in the pathogenesis of DR. Although its cellular consequences in endothelial cells, pericytes and neurons vary, the final results point to mitochondrial respiratory impairment, ROS accumulation, mtDNA damage, cellular apoptosis and progressive retinal neurovascular damage. Importantly, mitochondrial dynamics does not function in isolation. The damaged mitochondria produced by excessive division must be removed by mitophagy, and then the mitochondrial network must be reconstructed through mitochondrial biogenesis. Therefore, the interaction between fission–fusion balance, mitophagy and mitochondrial biogenesis is crucial to maintaining the homeostasis of retinal mitochondria (Liang et al., 2022).

### 2.2 Mitophagy dysfunction: impaired mitochondrial clearance in diabetic retinopathy

Mitophagy is a selective catabolic process that can remove mitochondria with abnormal function, which is crucial to MQC and cell homeostasis (Liu et al., 2024). It is mainly regulated through three interrelated pathways: PTEN-induced putative kinase 1 (PINK1)/Parkin-dependent pathway, receptor-mediated mitophagy and lipid-dependent mitophagy. These pathways work together to enable cells to identify, label and remove damaged mitochondria under physiological and stress conditions.

PINK1/Parkin pathway is the best-characterized mitophagy pathway at present. When the mitochondria lose the membrane potential, PINK1 will accumulate on the outer membrane of the mitochondria and recruit Parkin, thus promoting the ubiquitination of mitochondrial proteins. These ubiquitin signals recruit autophagy adaptor proteins, which directly bind to microtubule-associated protein 1 light chain 3 (LC3)/gamma-aminobutyric acid receptor-associated protein (GABARAP), thus leading to autophagosome formation and mitochondrial clearance (Li J. et al., 2023; Alia et al., 2026). At the same time, receptor-mediated mitophagy depends on mitochondrial receptors, such as B-cell lymphoma 2-interacting protein 3 (BNIP3), FUN14 domain containing 1 (FUNDC1) and PHB2, these receptors directly interact with LC3/GABARAP to initiate mitophagy and initiates mitochondrial degradation without ubiquitination (Shan et al., 2025). Lipid-mediated mitophagy is triggered by mitochondrial lipid remodeling, especially the accumulation of cardiolipin externalization or ceramide on the mitochondrial surface, which helps to engage autophagic machinery. These pathways work together and can compensate each other according to the stage of the disease and cellular stress.

In DR, mitophagy shows stage-dependent dysfunction. In the early stage of the disease, acute hyperglycemia stress can briefly activate mitophagy, especially through the PINK1/Parkin pathway to remove mildly damaged mitochondria. However, persistent high blood sugar and oxidative stress will gradually inhibit the core mitophagy protein and destroy the upstream regulatory signal. As a result, damaged mitochondria accumulate and become a continuous source of ROS and inflammatory stimulation (D'Amico et al., 2024). Under hypoxic conditions, the BNIP3/NIX pathway may provide partial compensation by enhancing mitochondrial clearance and reducing BRB damage (Wu et al., 2024). Similarly, lipid remodeling may have a protective effect in early DR, but the chronic accumulation and lipotoxicity of ceramides will affect the mitophagy flux and aggravate the damage to the retinal barrier (Shan et al., 2025).

This stage-dependent nature also gives mitophagy a double-edged sword in DR. In the early stage of the disease, moderately activated complete mitophagy flux can remove depolarized mitochondria and reduce the activation of inflammasomes, but chronic hyperglycemia may block autophagosome–lysosome fusion, resulting in mitophagy stagnation or ineffectiveness, even when LC3-II or PINK1/Parkin signaling is enhanced. On the contrary, under high metabolic stress, excessive mitophagy will exhaust functional mitochondria, weaken the vitality of endothelial cells and glial cells, and exacerbate the destruction of the BRB. Therefore, the end point of biological significance is not just “more mitophagy”, but the restoration of mitochondrial turnover at the right time and with normal flux.

Overall, mitophagy is a dynamic and sophisticated intracellular quality-control mechanism that governs the pathological progression of DR, with three regulatory pathways coordinating and compensating for each other throughout the stages of the disease. In particular, maintaining cellular homeostasis is based not only on the clearance of damaged mitochondria through mitophagy but also on the replenishment of functional mitochondria. As a complementary process to mitophagy, mitochondrial biogenesis generates new mitochondria to meet cellular energy demands and replace damaged organelles degraded under pathological stress.

### 2.3 Impaired mitochondrial biogenesis: a driver of bioenergetic collapse

Mitochondrial biogenesis is a core biological process that modulates mitochondrial quantity and function through multi-layered signaling cascades, transcriptional regulation, and nucleo-mitochondrial protein shuttling (Ji et al., 2021). PGC-1α serves as the master regulator of this process (Koh et al., 2021). In response to increased cellular energy demand, oxidative stress, and differentiation signals, PGC-1α translocates to the nucleus to activate nuclear respiratory factor 1 (NRF1) and other downstream transcription factors, thus upregulating mitochondrial transcription factor A (TFAM) expression (Fahed et al., 2022). Upon mitochondrial entry, TFAM facilitates mtDNA replication and transcription, stabilizes mtDNA structure, and promotes the synthesis of nuclear- and mitochondrial-encoded proteins to renew mitochondrial components. Mitochondrial biogenesis is tightly coordinated with mitochondrial dynamics and mitophagy to maintain MQC homeostasis. Newly generated mitochondria integrate into the mitochondrial network via fusion, while damaged mitochondria are eliminated through PINK1/Parkin-dependent mitophagy, and this synergistic regulation sustains normal mitochondrial quantity and cellular function (Hu et al., 2021; Hombrebueno et al., 2019). In DR, hyperglycemia disrupts mitochondrial biogenesis mainly by suppressing PGC-1α signaling. ROS overproduction, nuclear factor kappa B (NF-κB) activation, and accumulation of advanced glycation end-products inhibit PGC-1α expression and activity in retinal tissues, retinal microvascular endothelial cells, and Müller cells (Abu El-Asrar et al., 2025; Liu C. et al., 2025). Downregulation of PGC-1α reduces NRF1/2 and TFAM expression, thus altering mtDNA replication, mitochondrial transcription, and new mitochondrial synthesis (Chang et al., 2024). In early DR, insufficient biogenesis fails to compensate for hyperglycemia-induced mitochondrial damage and excessive mitophagy, resulting in reduced mitochondrial abundance, particularly in the outer retina. In advanced DR, persistent depletion of TFAM, instability of mtDNA, and reduced mtDNA copy number further aggravate mitochondrial failure (Hombrebueno et al., 2019).

However, PGC-1α should not be framed as an unconditionally protective target. In the early, bioenergetically compromised retina, activation of the PGC-1α/NRF1/TFAM axis may support mtDNA maintenance and mitochondrial renewal. In contrast, In the hypoxic and pro-angiogenic environment of PDR, the PGC-1α/ERR-α transcriptional program has been associated with increased VEGF and angiopoietin 2 expression. Indiscriminate PGC-1α activation could therefore theoretically reinforce pathological neovascularization (Abu El-Asrar et al., 2025). Therefore, future mechanistic studies should distinguish NRF1/TFAM-mediated mitochondrial biogenesis from ERR-α-driven angiogenic transcription and define a stage-specific therapeutic window.

A key feature of DR is metabolic memory, in which mitochondrial injury and retinal dysfunction continue even after glycemic control is restored. Epigenetic modifications, including DNA methylation and histone deacetylation at the PGC-1α promoter, can persistently repress PGC-1α transcription and thereby maintain mitochondrial biogenesis defects (Li B. et al., 2024). Long noncoding HOX transcript antisense intergenic ribonucleic acid (HOTAIR) has also been implicated in this process. High glucose induces sustained upregulation of HOTAIR in retinal vascular and non-vascular cells (Kumar and Kowluru, 2026). Acting as an epigenetic scaffold, HOTAIR recruits polycomb-repressive complex 2 and other histone-modifying complexes to silence mitochondrial biogenesis-related genes, including deoxyribonucleic acid polymerase gamma and Twinkle helicase. These effects may persist under normoglycemic recovery conditions in retinal endothelial cells and Müller cells, providing a molecular basis for metabolic memory of DR.

Mitochondrial biogenesis defects link metabolic stress induced by hyperglycemia to retinal neurovascular damage. When damaged mitochondria cannot be replaced by new functional mitochondria, dysfunctional mitochondria will accumulate, resulting in cellular energy exhaustion in the NVU. This lack of energy will promote oxidative stress, the release of inflammatory mediators and apoptotic signaling. Therefore, the integrity of blood vessels is damaged, the loss of pericytes is accelerated, retinal neuroinflammation is aggravated, and retinal ganglion cells and microvascular endothelial cells are more vulnerable to damage. Abnormal downregulation of mitochondrial ribosomal protein S21 may damage mitochondrial translation and ribosomal function, and MQC dysfunction and retinal metabolic damage (Zhang Y. et al., 2026). The consequences of impaired mitochondrial biogenesis are cell type-specific. In retinal microvascular endothelial cells, mitochondrial biogenesis defects mainly damage the integrity of the barrier and increase vascular permeability. In Müller cells, it disrupts glutamate metabolism and lactate transport, thus indirectly aggravating neuronal damage. Photoreceptors have high mitochondrial density and metabolic needs, and are particularly sensitive to mitochondrial biogenesis defects; impaired mitochondrial renewal in these cells may lead to early visual decline in DR (Miller et al., 2020). Other pathways further amplify the process. For example, hyperglycemia inhibits SIRT3 activity, thus reducing PGC-1α function and promoting the accumulation of ROS, forming a vicious cycle of mitochondrial damage (Li and Cai, 2022). Hyperglycemia combined with hyperhomocysteinemia can also accelerate the production of ROS and mtDNA damage in retinal endothelial cells, damaging mitochondrial respiration, biogenesis and mitophagy. Hydrogen sulfide donor GYY4137 can reduce these injuries and support the protective role of homocysteine-hydrogen sulfide homeostasis in early DR intervention (Malaviya and Kowluru, 2024).

In summary, inhibiting mitochondrial biogenesis is a key pathological mechanism that induces retinal metabolic disorders, destroys MQC homeostasis and causes retinal neurovascular damage. This dysfunction will continue to exist during the progression of DR, destroying retinal energy metabolism and aggravating oxidative stress and inflammatory reactions. Therefore, targeting the PGC-1α pathway to restore mitochondrial biogenesis and rebalance MQC homeostasis is a therapeutic strategy that is expected to prevent or slow DR progression.

### 2.4 Emerging pathways

In addition to mitochondrial dynamics, mitophagy, and mitochondrial biogenesis, several emerging pathways contribute to MQC disruption in DR and may provide additional therapeutic targets. Mitochondria-associated endoplasmic reticulum membranes (MAMs) are dynamic contact sites between the endoplasmic reticulum and mitochondria. They regulate Ca^2+^ transfer, lipid exchange, mitochondrial dynamics, endoplasmic reticulum stress, autophagy, apoptosis, and inflammation (Zhang et al., 2019; Wang et al., 2025). Their function depends on tethering complexes such as inositol 1,4,5-trisphosphate receptor type 1 (IP3R1)-glucose-regulated protein 75 (GRP75)-voltage-dependent anion channel 1 (VDAC1), B-cell receptor-associated protein 31 (BAP31)-mitochondrial fission 1 protein (FIS1), MFN2, and vesicle-associated membrane protein-associated protein B (VAPB)-protein tyrosine phosphatase-interacting protein 51 (PTPIP51), which coordinate inter-organelle signaling and material exchange (Ding et al., 2024). In DR, hyperglycemia alters the structure and function of MAMs, leading to calcium overload, oxidative stress, neuroinflammation, and vascular dysfunction (Gao et al., 2020). Dysregulation of the IP3R1-GRP75-VDAC1 axis enhances mitochondrial Ca^2+^ entry, promotes mitochondrial permeability transition pore opening, increases ROS production, and facilitates apoptosis in retinal microvascular endothelial cells (Yang and Yang, 2025). Calcium overload may also activate the cyclic GMP-AMP synthase (cGAS)-stimulator of interferon genes (STING) pathway and exacerbate Müller cell-mediated neuroinflammation. In addition, altered FUNDC1-inositol 1,4,5-trisphosphate receptor type 2 (IP3R2) coupling can impair endothelial calcium signaling and disturb vascular endothelial growth factor receptor 2-related angiogenic responses. These findings suggest that targeting MAMs tethering complexes or restoring MAMs homeostasis may represent a promising therapeutic strategy for DR.

Intercellular mitochondrial transfer, also known as horizontal mitochondrial transfer, enables damaged recipient cells to acquire functional mitochondria from donor cells, thereby improving respiration and survival (Soman et al., 2025). This process is mediated mainly by tunneling nanotubes (TNTs), gap junctions, and extracellular vesicles (Zamberlan and Semenzato, 2025). Among these routes, TNT-mediated transfer has been studied most extensively in ocular and retinal cell communication. TNTs are F-actin-based membrane channels, and their formation is influenced by factors such as tumor necrosis factor alpha-induced protein 2, platelet-derived growth factor-BB, Rho-associated coiled-coil containing protein kinase signaling, and the mitochondrial motor adaptor mitochondrial Rho guanosine triphosphatase 1 (Jiang et al., 2020; Yuan et al., 2024). Mitochondrial transfer is often directional and cell-type selective; for example, pericyte-to-endothelial mitochondrial transfer appears to occur predominantly in one direction (Kempf et al., 2024). In the DR microenvironment, this protective transfer system is impaired. Pericyte loss reduces the availability of mitochondrial donor cells, while abnormal pericyte morphology disrupts functional transfer units. In diabetic Ins2Akita mice, the coverage of retinal pericytes decreases substantially, although the number and length of TNTs may increase as a compensatory response (Kempf et al., 2024). In addition, the lipid mediator 19,20-dihydroxydocosapentaenoic acid, which is elevated in the diabetic retina, directly inhibits the formation of TNTs and weakens the transfer of pericyte-endothelial mitochondria. This blockade reduces ROS clearance and limits metabolic rescue of damaged endothelial cells. Proteomic analyses further indicate that 19,20-dihydroxydocosapentaenoic acid alters mitochondrial proteins, platelet-derived growth factor receptor beta-related signaling, and transport-associated proteins at the pericyte-endothelial interface, thus disrupting PI3K-Akt, PDGFR-β, and RhoA signaling without directly affecting early platelet-derived growth factor receptor phosphorylation (Kempf et al., 2024).

Mitochondrial-derived vesicles (MDVs) are subcellular structures that bud directly from the mitochondrial membrane (Oh et al., 2023). Their diameter is usually 70–150 nm, and they may contain either a single or double membrane. They selectively package mitochondrial components, including mtDNA, proteins, and lipids, but do not constitute complete mitochondria (Gui et al., 2025). MDVs differ from extracellular vesicles, which are membrane-bound vesicles secreted by cells, usually 40–160 nm in diameter, and originate from the plasma membrane or multivesicular bodies. MDVs arise directly from mitochondria and retain membrane integrity and partial mitochondrial function (Ore et al., 2024). They participate in the removal of mitochondrial components and in the repair of mitochondrial function. They can selectively package oxidized proteins or lipids and deliver them to lysosomes for degradation, thereby alleviating mitochondrial stress (Na et al., 2025). MDVs can also serve as signaling carriers that transmit mitochondria-derived molecules to other organelles or extracellular spaces to regulate cellular stress responses (Rapushi et al., 2026). By preserving the integrity of the mitochondrial membranes and components of the electron transport chain, MDVs retain a limited capacity for ATP synthesis. After coenzyme Q10 modification, this capacity is significantly enhanced, thereby improving their bioenergetic function. In DR, MDVs show favorable stability and therapeutic potential (Gui et al., 2025). Endosomal mitochondrial-derived vesicles (eMDVs) retain key mitochondrial structural proteins, including TOMM20, VDAC1, and OPA1, as well as certain electron transport chain complexes, conferring strong antioxidant and bioenergetic properties. After local administration, eMDVs can effectively penetrate the BRB and selectively accumulate in the retinal microvasculature, indicating excellent retinal targeting. In addition, eMDVs effectively regulate the integrated stress response (ISR) by inhibiting the eIF2α-ATF4-CHOP signaling pathway, thereby reducing endoplasmic reticulum and mitochondrial stress induced by high glucose and restoring cellular homeostasis. eMDVs also significantly enhance mitochondrial membrane potential, ATP synthesis capacity, and mtDNA copy number, while reducing ROS levels and increasing the activity of antioxidant enzymes such as superoxide dismutase (SOD) and glutathione peroxidase. Together, these properties suggest that MDVs may offer advantages over conventional exosomes as nano-delivery platforms.

### 2.5 Mitochondrial quality control network in the early and late stages of diabetic retinopathy

**FIGURE 3:**
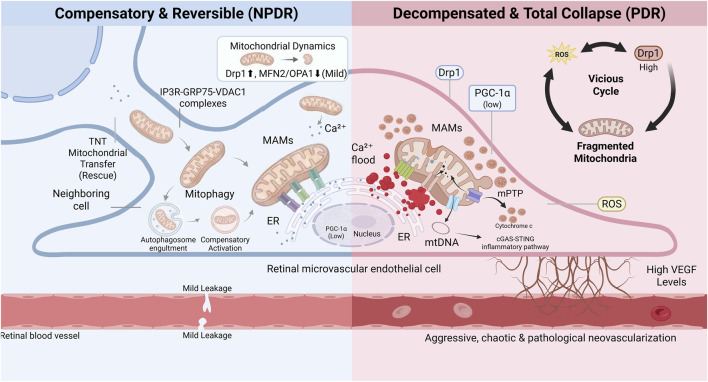
Compensatory and decompensatory mechanisms of mitochondrial quality control in retinal microvascular endothelial cells during diabetic retinopathy progression. The diagram illustrates the transition from the early, reversible stage of NPDR to the late, decompensated and collapsed stage of PDR. In the NPDR phase, mild disturbances in mitochondrial dynamics are counteracted by protective pathways including mitophagy and mitochondrial transfer via tunneling nanotubes, facilitated by MAMs and controlled calcium signaling, resulting in only mild vascular leakage. As the disease progresses to PDR, excessive Drp1-driven mitochondrial fragmentation triggers a destructive vicious cycle characterized by a massive burst of ROS and a calcium flood from the ER, which leads to mPTP opening and the release of cytochrome c and mtDNA. This cascade activates the cGAS-STING inflammatory pathway, driving high VEGF levels and ultimately resulting in aggressive, chaotic pathological neovascularization. The diagram highlights the critical role of mitochondrial quality control breakdown in driving diabetic retinopathy progression.

Mitochondrial dynamics, mitophagy, and mitochondrial biogenesis form an MQC network centered on damage recognition, targeted removal, and functional compensation, operating through interactions among core molecules and coupled signaling pathways (Figure 3). Under physiological conditions, these three processes form a closed regulatory loop that maintains mitochondrial morphological and functional homeostasis in retinal cells. MAMs, as a central hub for intercellular signaling and substance exchange, also participate in the regulation of MQC by modulating key processes such as calcium homeostasis and lipid transport. As a repair mechanism, intercellular mitochondrial transfer supplies healthy mitochondria to damaged retinal cells and therefore constitutes an important supplementary component of the MQC network. During the pathological progression of DR from early to advanced stages, the MQC network shifts from a compensatory and reversible mild imbalance to a decompensated and irreversible collapse, ultimately mediating progressive injury to retinal microvascular and neural cells.

The core features of NPDR include hyperglycemia-induced metabolic disturbances, mild oxidative stress, and injury to microvascular endothelial cells and pericytes (Huang et al., 2026). At this stage, the MQC imbalance is mild, compensatory, and localized, manifesting primarily as abnormal mitochondrial dynamics, mitophagy activation, delayed mitochondrial biogenesis, mild MAM dysfunction, and partial mitochondrial-transfer rescue (Chen et al., 2025). Under sustained high-glucose conditions, mitochondrial dynamics become imbalanced in retinal microvascular endothelial cells, pericytes, and other retinal cells: the key fission regulator Drp1 is excessively activated, whereas the expression of fusion regulators such as MFN2 and OPA1 decreases, causing mild mitochondrial fragmentation (Mohammad and Kowluru, 2022). A small number of damaged mitochondria act as signals that trigger mitophagy, during which the three major mitophagy pathways are compensatorily activated to initiate protective responses aimed at removing damaged mitochondria and restoring retinal cellular homeostasis. Concurrently, high-glucose-induced mild oxidative stress inhibits autophagosome-lysosome fusion, leading to impaired autophagic flux and incomplete degradation of damaged mitochondria (Yang et al., 2019). ROS generated by abnormal mitochondrial dynamics would normally initiate mitochondrial biogenesis through the adenosine monophosphate-activated protein kinase (AMPK) pathway as a compensatory response; however, hyperglycemia transiently activates the mechanistic target of rapamycin (mTOR) pathway, suppressing the transcriptional activity of PGC-1α, NRF1, and TFAM, thereby delaying compensatory mitochondrial biogenesis (Nanjaiah and Vallikannan, 2019). MAMs exhibit excessive coupling and mild functional disturbance. This causes mitochondrial calcium overload, mtDNA release, ER stress, and protein misfolding, which can promote apoptosis, inflammation, and BRB disruption (Zhang et al., 2024). If high-glucose stress is relieved promptly, the MQC system may restore homeostasis through self-regulation.

In contrast, the core features of PDR include severe retinal ischemia and hypoxia, extensive inflammation, and neovascularization (Huang et al., 2026). Persistence of the high-glucose microenvironment drives the MQC network from an early compensatory local imbalance to irreversible global dysfunction characterized by collapse of mitochondrial dynamics, mitophagy deficiency, mitochondrial-biogenesis inactivation, profound MAMs disruption, and failure of compensatory mitochondrial transfer. Together, these five components form a persistent vicious cycle that ultimately causes irreversible damage to retinal cells. Damaged mitochondria that are not fully cleared in early DR continue to accumulate, accompanied by progressively increasing ROS levels. ROS exacerbate mitochondrial-dynamics collapse by further upregulating Drp1 and suppressing MFN2 and OPA1, leading to severe mitochondrial fragmentation. In parallel, ROS and accumulated damaged mitochondria inhibit major mitophagy pathways, shifting mitophagy from compensatory activation to functional deficiency and creating a vicious cycle (Wei et al., 2026). With continuous accumulation of damaged mitochondria, the expression of PGC-1α, NRF1, and TFAM decreases markedly, and mitochondrial biogenesis becomes completely inactivated. At this stage, profound structural and functional disruption of MAMs becomes a key node that aggravates MQC collapse and promotes PDR progression: abnormal alterations in MAM spacing abolish normal Ca^2+^ exchange and lipid transport; the stability of anchoring protein complexes is disrupted; and the IP3R1-GRP75-VDAC1 axis becomes severely dysfunctional, leading to uncontrolled Ca^2+^ transport (Li et al., 2022). Excess Ca^2+^ entry into mitochondria triggers calcium overload, opens the mitochondrial permeability transition pore, further promotes ROS production and cytochrome c release, and accelerates apoptosis of retinal microvascular endothelial cells. Meanwhile, calcium overload activates the cGAS-STING pathway, exacerbates Müller cell-mediated chronic neuroinflammation, and disrupts retinal NVU homeostasis (Li Y. et al., 2023). Severe MAMs disruption can no longer be reversed by self-regulation and further amplifies abnormalities in mitochondrial dynamics, mitophagy, and mitochondrial biogenesis, thereby accelerating network collapse and establishing an irreversible vicious cycle that culminates in complete retinal homeostatic failure.

Although this framework over the course of the disease is useful, it should not be interpreted as following a rigid linear sequence in each area of the retina. Endothelial cells may have barrier dysfunction and pathological angiogenic signaling, while pericytes may have early mitochondrial fission-mediated apoptosis. Müller cells may exacerbate glial inflammation and change metabolic support, while RGCs and RPE cells may have differences in neurodegenerative vulnerability and lysosomal reserve. These cell type differences mean that the same MQC intervention may restore homeostasis in one region, while the benefit may be limited or even risky in another region.

## 3 Orchestrating mitochondrial quality control: natural products as multitargeted agents for remodeling retinal homeostasis

**FIGURE 4:**
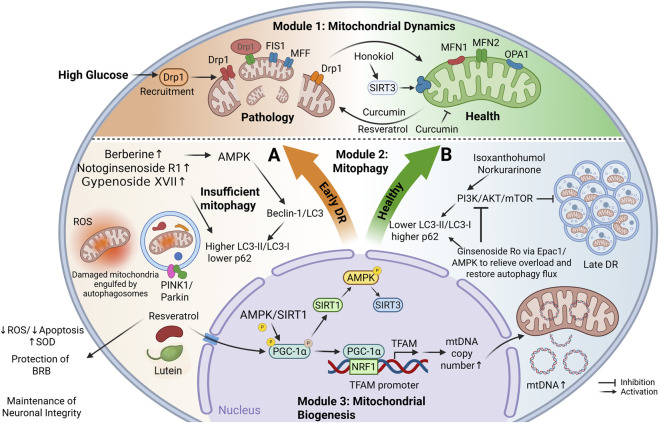
Mechanisms by which natural products regulate the mitochondrial quality control network in the treatment of diabetic retinopathy. The diagram illustrates that, in high-glucose-induced injury to retinal microvascular endothelial cells, NPs restore mitochondrial function through three key modules: mitochondrial dynamics, mitophagy, and mitochondrial biogenesis. Specifically, they inhibit Drp1-mediated excessive fission and promote fusion via the SIRT3/MFN1/MFN2/OPA1 axis, restore mitophagic flux by modulating the AMPK/mTOR pathway (distinguishing between early and late DR states), and activate the PGC-1α/TFAM/NRF1-dependent biogenesis pathway to increase mtDNA copy number. These actions ultimately alleviate oxidative stress, reduce apoptosis, elevate SOD levels, and protect the BRB, thereby supporting natural product-based therapeutic strategies for the treatment of DR.

NPs derived from plants, fungi and microorganisms are rich in a variety of chemical structures and bioactive compounds with favorable pharmacological properties, making them valuable lead compounds for therapeutic drug research and development (Cui and Kim, 2024). In the study of DR, the main bioactive NPs categories include polyphenols, flavonoids, saponins and alkaloids. Compared with synthetic single-target small molecule drugs, these compounds have significant structural diversity, low cytotoxicity and excellent biocompatibility (Li et al., 2025). Functionally, they exert multitarget antioxidant, anti-inflammatory, neuroprotective and metabolic regulatory effects (Suswidiantoro et al., 2026). This multifaceted activity allows them to intervene at multiple pathological nodes of chronic metabolic disease rather than acting on a single downstream effector. Unlike conventional clinical therapies for DR, which primarily alleviate late-stage vascular lesions, NPs may penetrate retinal tissue, target the retinal NVU, and modulate mitochondrial homeostasis more broadly. Increasing evidence indicates that multiple NPs can regulate core MQC modules, including mitochondrial dynamics, mitophagy, and mitochondrial biogenesis, thereby counteracting hyperglycemia-induced mitochondrial dysfunction and NVU impairment. These effects may help restore retinal homeostasis and attenuate the progression of DR. The following section summarizes the multitarget mechanisms by which representative NPs remodel MQC network balance in DR. These multitarget effects are particularly relevant because mitochondrial dynamics, mitophagy, and biogenesis function as interconnected MQC modules rather than isolated pathways (Figure 4; Table 1).

**TABLE 1:** Natural products involved in the treatment of diabetic retinopathy by targeting core hubs in the mitochondrial quality control network.

Target	Classification	Natural product	Model	Dose	Application	Molecular mechanism	Molecular target(s)	Therapeutic outcome	References
Mitochondrial Dynamics	Polyphenols	Honokiol	db/db mice; Rat retinal microvascular endothelial cells	0.4 mg/kg; 10, 30 μM	*In vivo; In vitro*	Activates SIRT3, a key regulator of mitochondrial homeostasis, thereby upregulating the expression of the mitochondrial fusion protein OPA1, reversing high glucose-induced excessive mitochondrial division and fragmentation	SIRT3 ↑, OPA1 ↑	Reverses excessive mitochondrial division and fragmentation	Shi et al. (2024)
​	​	Resveratrol	Adult zebrafish	5, 50 mg/L	*In vivo*	Activates SIRT1 and mitochondrial deacetylases, regulating the dynamic balance of the fusion protein OPA1 and the fission protein FIS1, enhancing mitochondrial DNA repair, thereby maintaining mitochondrial homeostasis and exerting retinal neuroprotective effects	SIRT1 ↑, OPA1 ↑, FIS1 ↓	Retinal neuroprotection, maintains mitochondrial homeostasis	Sheng et al. (2018)
​	​	Curcumin	Rat	5 μM	*In vitro*	Downregulates Drp1 and upregulates MFN2 to inhibit excessive mitochondrial fission and promote fusion, stabilizing mitochondrial morphology and function, reducing ROS production and mitochondrial membrane potential disruption, thereby inhibiting abnormal mitophagy and apoptosis	Drp1 ↓, MFN2 ↑	Reduces ROS and mitochondrial membrane potential disruption, inhibits abnormal mitophagy and apoptosis	Xu et al. (2022)
Mitophagy	Terpenoids	Gypenoside XVII	db/db mice	10, 20 mg/kg	*In vivo*	Reduces Bax and Caspase-3 expression, while increasing Bcl-2 expression, thereby exerting anti-apoptotic effects; enhances autophagy-related proteins such as ATG5, Beclin1, and the LC3-II/LC3-I ratio, and reduce p62 protein expression, resulting in pro-autophagy effects	Bax ↓, Caspase-3 ↓, Bcl-2 ↑, ATG5 ↑, Beclin1 ↑, LC3-II/LC3-I ↑, p62 ↓	Anti-apoptotic effects, pro-autophagy effects	Luo et al. (2021)
​	​	Notoginsenoside R1	db/db mice; Müller cells	30 mg/kg; 5, 10, 20, 40 µM	*In vivo; In vitro*	Activates the PINK1/Parkin axis, promoting autophagosome-lysosome colocalization	PINK1/Parkin axis ↑	Promotes autophagosome-lysosome colocalization	Zhou et al. (2019)
​	​	Ginsenoside Ro	Mouse; Human retinal microvascular endothelial cells	90 mg/kg; 6.25, 12.5 μM	*In vivo; In vitro*	Increases Epac1 protein expression and AMPK phosphorylation levels, indicating the activation of the Epac1/AMPK signaling pathway; reduces the number of AGE-induced autolysosomes, decreases PINK1 and Parkin expression levels, and reduces mitophagy	Epac1 ↑, AMPK ↑, PINK1 ↓, Parkin ↓	Reduces AGE-induced autolysosomes, reduces mitophagy	Liu J. et al. (2025)
​	Flavonoids	Norkurarinone & Isoxanthohumol	Human retinal microvascular endothelial cells	20, 40, 80 μM	*In vitro*	Activate the PI3K/AKT/mTOR survival pathway, thereby negatively regulating Beclin-1/ATG5	PI3K/AKT/mTOR ↑, Beclin-1 ↓, ATG5 ↓	Suppresses autophagy (via survival pathway)	Zhao et al. (2022)
​	Alkaloids	Berberine	Müller cells	2.5, 5, 10, 20 µM	*In vitro*	Dependent on the AMPK activation pathway, it upregulates the adapter proteins LC3 and Beclin-1	AMPK ↑, LC3 ↑, Beclin-1 ↑	Promotes autophagy	Chen et al. (2018)
Mitochondrial Biogenesis	Polyphenols	Resveratrol	Rat retinal capillary endothelial cells; STZ-induced rat	10–50 μM; 10 mg/kg/day	*In vitro; In vivo*	Activates the AMPK/SIRT1/PGC-1α signaling pathway, promotes mitochondrial DNA transcription and replication, upregulates mitochondrial transcription factor A expression, thereby enhancing mitochondrial biogenesis and improving mitochondrial function. Concurrently, it reduces ROS generation and inhibits high glucose-induced apoptosis of retinal capillary endothelial cells	AMPK/SIRT1/PGC-1α ↑, TFAM ↑	Enhances mitochondrial biogenesis, reduces ROS, inhibits apoptosis	Delmas et al. (2021)
​	Terpenoids	Lutein	ARPE-19; STZ-induced hyperglycemic Wistar rats	10 μM; -	*In vitro; In vivo*	Activates AMPK phosphorylation, initiating upstream signals for mitochondrial biogenesis, thereby upregulating PGC-1α mRNA and protein expression, and activating transcription of its downstream target genes NRF1 and TFAM. Significantly increases mtDNA copy number in diabetic models, restoring mitochondrial biogenic capacity; simultaneously, it reduces ROS levels and alleviates oxidative stress, thus protecting mitochondrial structural integrity and mtDNA stability	AMPK ↑, PGC-1α ↑, NRF1 ↑, TFAM ↑	Increases mtDNA copy number, reduces ROS, protects mitochondrial structure and mtDNA stability	Nanjaiah and Vallikannan (2019)

### 3.1 Targeting mitochondrial dynamics: polyphenols as modulators of fission-fusion homeostasis in diabetic retinopathy

Under high-glucose conditions, mitochondrial fission-fusion balance is disrupted, typically with increased expression of fission-related proteins such as Drp1 and decreased expression of fusion-related proteins such as MFN1, MFN2, and OPA1. This imbalance promotes excessive mitochondrial fission and fragmentation, thereby impairing mitochondrial function. NPs may counteract this pathological shift by directly or indirectly regulating key dynamics proteins.

The polyphenol honokiol restores mitochondrial dynamics indirectly by modulating upstream energy and stress sensors. *In vivo* and *in vitro* experiments using db/db mice and rat retinal microvascular endothelial cells showed that honokiol, at 0.4 mg/kg or 10 and 30 μM, activated SIRT3, a key regulator of mitochondrial homeostasis, thereby upregulating OPA1 expression and reversing high-glucose-induced mitochondrial fission and fragmentation. This significantly improved mitochondrial function, reduced oxidative stress, and inhibited apoptosis of retinal microvascular endothelial cells, ultimately alleviating vascular leakage and microvascular injury in DR (Shi et al., 2024). Another polyphenol, resveratrol, acts more broadly by regulating both the expression and the activity of key proteins. *In vivo* studies in adult zebrafish showed that resveratrol at 5 or 50 mg/L, through modulation of SIRT1 and AMPK, not only downregulated the fission-related proteins Drp1, FIS1, and mitochondrial fission factor, but also upregulated the fusion-related proteins MFN1 and MFN2 (Sheng et al., 2018). This bidirectional regulation restored mitochondrial homeostasis, reduced ROS generation, and inhibited retinal cell apoptosis. Curcumin exerts similar effects by rebalancing the expression of core mitochondrial dynamics proteins. *In vitro* studies showed that 5 µM curcumin inhibited excessive mitochondrial fission and promoted fusion by downregulating Drp1 and upregulating MFN2 (Xu et al., 2022). This stabilized mitochondrial morphology and function, reduced ROS production and loss of mitochondrial membrane potential, and thus inhibited abnormal mitophagy and apoptosis, ultimately protecting retinal neurons from oxidative stress and improving DR.

In general, although different polyphenolic NPs regulate mitochondrial dynamics through distinct pathways, they all exert protective effects against high-glucose-induced retinal injury by restoring the balance between fission and fusion and improving mitochondrial function.

### 3.2 Correcting autophagy imbalance: context-dependent regulation of mitophagy by natural products

Mitophagy is a central hub of the MQC network and a key mechanism for the selective removal of damaged mitochondria. In early DR, insufficient autophagy may impair MQC and limit the clearance of damaged cellular components, contributing to chronic retinal cell injury and dysfunction. In advanced disease, however, excessive autophagy may aggravate pathological damage by promoting self-digestion, BRB injury, and neovascularization. Thus, NP-based regulation of mitophagy in DR is highly context-dependent and requires stage-specific modulation.

Under conditions of insufficient autophagy, notoginsenoside R1 enhances mitophagy by upregulating PINK1 and Parkin expression, increasing the LC3-II/LC3-I ratio, promoting colocalization of mitochondria and autophagosomes, and reducing p62 levels (Zhou et al., 2019). This facilitates the clearance of damaged mitochondria and reduces oxidative stress and inflammation. In an *in vivo* study in db/db mice, gypenoside XVII at 10 and 20 mg/kg not only initiated autophagy but also promoted autolysosome formation by increasing autophagy-related protein 5, Beclin-1, and the LC3-II/LC3-I ratio, while lowering p62 levels (Luo et al., 2021). Improved autophagosome-lysosome fusion enhanced autophagic flux and accelerated the removal of damaged mitochondria, thereby helping restore intracellular homeostasis in retinal cells. *In vitro* experiments further confirmed that berberine (2.5–20 µM) activated AMPK, increased Beclin-1 and LC3 levels in Müller cells, and reduced apoptosis (Chen et al., 2018). Pharmacological inhibition of AMPK abolished this protective effect, confirming that berberine acts through the AMPK pathway. By contrast, under conditions of pathological autophagy overactivation, *in vivo* and *in vitro* models using murine and human retinal microvascular endothelial cells showed that ginsenoside Ro (90 mg/kg; 6.25 and 12.5 μM) moderately inhibited abnormally elevated PINK1/Parkin signaling through the Epac1/AMPK pathway (Liu J. et al., 2025). This reduced autophagosome accumulation and restored autophagic flux. The accompanying changes in LC3-II and p62 were consistent with restoration of autophagic homeostasis. Flavonoids such as norkurarinone and isoxanthohumol also modulate this process by moderately activating the phosphoinositide 3-kinase (PI3K)/protein kinase B (AKT)/mTOR pathway (Zhao et al., 2022). This reduces ROS, stabilizes mitochondrial membrane potential, inhibits autophagosome formation, lowers Beclin-1 and autophagy-related protein 5 levels, decreases the LC3-II/LC3-I ratio, and increases p62, changes that are consistent with reduced autophagy-dependent cell death.

In summary, NP-mediated regulation of mitophagy in DR is complex and strongly context-dependent. Notoginsenoside R1 and gypenoside XVII enhance mitophagy and promote clearance of damaged mitochondria under conditions of insufficient autophagy. Berberine restores AMPK-dependent autophagic activity, whereas ginsenoside Ro, norkurarinone, and isoxanthohumol help restrain pathological autophagy overactivation and re-establish autophagic balance.

### 3.3 Reigniting mitochondrial biogenesis: natural products act through the AMPK-sirt1-pgc-1α axis

As discussed in the previous section, PGC-1α activation carries a stage-dependent duality: beneficial in early bioenergetically compromised retinas, but potentially detrimental in the hypoxic, pro-angiogenic environment of PDR via the ERR-α/VEGF axis. Therefore, the following evaluation of NPs focuses on NPDR models where mitochondrial biogenesis failure dominates and angiogenesis is not yet a concern.

Promoting mitochondrial biogenesis is essential for rebuilding the mitochondrial network. This process is primarily governed by the PGC-1α/NRF1/TFAM transcriptional cascade. Accordingly, NPs can exert therapeutic effects in DR by activating this pathway. Resveratrol activates the AMPK and SIRT1 signaling pathways, thus synergistically regulating the core coactivator PGC-1α and upregulating the expression of NRF1 and TFAM (Delmas et al., 2021). This promotes mtDNA replication and transcription, enhances mitochondrial biogenesis, and improves mitochondrial function. Through these mechanisms, resveratrol exerts multiple protective effects in the retina: it reduces ROS levels, increases SOD activity in retinal tissue, and suppresses the mitochondrial apoptotic pathway, as reflected by downregulation of Bax, reduced cytochrome c release, and decreased caspase-3 activation (Biju and Andavar, 2025). Together, these effects protect retinal cells and delay the progression of DR. *In vitro* and *in vivo* models using ARPE-19 cells and STZ-induced hyperglycemic Wistar rats showed that lutein and its oxidative derivatives, at 10 μM, activate AMPK phosphorylation and provide an upstream signal for mitochondrial biogenesis (Nanjaiah and Vallikannan, 2019). These compounds subsequently upregulate PGC-1α at both the mRNA and protein levels and induce transcriptional activation of its downstream target genes, NRF1 and TFAM. In diabetic models, treatment with these NPs significantly increased mtDNA copy number, as determined by the mitochondrial nicotinamide adenine dinucleotide dehydrogenase 1/hypoxanthine phosphoribosyltransferase ratio, indicating restoration of mitochondrial biogenesis. This process also reduced ROS levels and oxidative stress, thereby protecting mitochondrial structural integrity and preserving mtDNA stability.

In brief, NPs such as resveratrol and lutein promote mitochondrial biogenesis by activating upstream AMPK-related signaling. Resveratrol activates PGC-1α through the AMPK/SIRT1 axis while also suppressing the Bax/caspase-3 apoptotic pathway and enhancing SOD activity, indicating dual actions on mitochondrial regeneration and cell survival. By contrast, Lutein mainly enhances mitochondrial renewal and antioxidant defense. Although their mechanisms differ, both contribute to restoration of mitochondrial biogenesis and may serve as promising therapeutic candidates for DR.

## 4 Clinical translation of natural products: pharmacokinetics and nano-delivery

Although NPs have putative advantages in multitarget regulation and relatively low toxicity when used to remodel the MQC network in DR, their clinical translation still faces substantial barriers. The pharmacokinetic data summarized in Table 2 help explain why the *in vitro* mechanisms described in Section 3 have not yet translated into clear clinical efficacy (Table 2). The main bottlenecks lie in pharmacokinetic limitations and potential toxicities, whereas emerging drug-delivery systems may provide opportunities to overcome these barriers. The following sections discuss these limitations and the corresponding strategies in greater detail.

**TABLE 2:** Summary of pharmacokinetic data for natural products intervening in diabetic retinopathy.

Classification	Natural product	Model	Route	Dose	T_max_ (h)	C_max_ (ng/mL)	AUC (0- ∞ ) (ng·h/mL)	T_1/2_ (h)	CL	References
Polyphenols	Honokiol	Type 2 Diabetic SD Rat	i.g	50 mg/kg	0.31 ± 0.11	614.7 ± 182.4	3,437.7 ± 534.1	3.84 ± 0.54	-	​
​	Resveratrol	Healthy Male	p.o	200 mg/d	0.5–1.5	23.5 ± 7.4	56.1 ± 35.1	3.6 ± 2.4	56.4 ± 25.3 L/h/kg	Nunes et al. (2009)
​	​	Healthy Female	p.o	200 mg/d	0.5–3	26.3 ± 14.5	51.2 ± 27.5	3.1 ± 1.5	71.52 ± 30.78 L/h/kg	Zheng et al. (2016)
​	Curcumin	STZ-induced Diabetic Rat	p.o	500 mg/kg	0.25 ± 0.00	60 ± 10	-	0.545 ± 0.215	51.0 ± 14.4 L/h/kg	Gutierres et al. (2015)
Terpenoids	Lutein	STZ-induced Diabetic Rat	i.g	600 μM	4	15.25 ± 1.6	120.4 ± 7.6 (0–16 h)	4.06 ± 0.2	0.54 ± 0.01	Toragall and Baskaran (2021)
Alkaloids	Berberine	Rat	i.v	4.0 mg/kg	-	963.00 ± 258.00	963,000 ± 134,000	23.60 ± 6.53	0.021 ± 0.003 L/h/kg	Feng et al. (2020)
​	Berberine	Rat	p.o	48.2 mg/kg	2.75 ± 2.95	4.11 ± 0.53	31,400 ± 3,010	5.74 ± 4.60	1.54 ± 0.14 L/h/kg	​

### 4.1 Pharmacokinetic barriers: bridging bioavailability and retinal delivery

**FIGURE 5:**
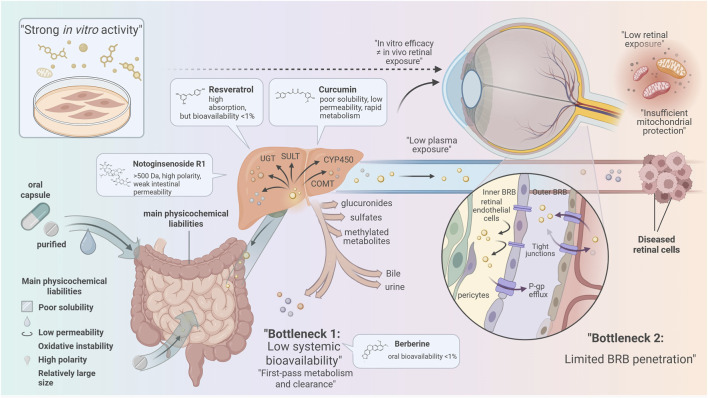
Bottlenecks in oral delivery and retinal penetration of natural products for the treatment of diabetic retinopathy. The diagram illustrates that after oral administration, NPs (e.g., resveratrol, curcumin, berberine) suffer from low systemic bioavailability primarily due to poor physicochemical properties (e.g., poor solubility, high polarity) and extensive first-pass metabolism by hepatic enzymes (e.g., UGT, CYP450), leading to rapid systemic clearance. Furthermore, the minimal fraction that reaches the systemic circulation is hindered by the physical and biological barriers of the BRB—including tight junctions and P-glycoprotein (P-gp) efflux pumps—making it extremely difficult to achieve therapeutic concentrations in the target retinal cells and their mitochondria. This creates a core challenge of “potent *in vitro* activity but insufficient retinal exposure *in vivo*,” providing a strong rationale for the development of targeted delivery strategies.

Numerous experimental studies have shown that a variety of NPs can inhibit DR progression in both *in vivo* and *in vitro* models. However, a major obstacle to clinical translation is the marked discrepancy between effective concentrations observed *in vitro* and the plasma concentrations actually achieved *in vivo*. For most NPs, the principal challenges are low systemic bioavailability and limited penetration across the BRB. Systemic bioavailability determines how much drug reaches the circulation, whereas BRB permeability determines whether the compound can access retinal tissue. Together, these factors substantially constrain the clinical translation and efficacy of NPs (Figure 5). This concentration-exposure mismatch is not merely a pharmacokinetic inconvenience but a major evidence limitation. For compounds such as curcumin, berberine, resveratrol, and notoginsenoside R1, effective *in vitro* concentrations often exceed expected free retinal exposure after oral administration. Therefore, retinal tissue levels, unbound concentrations, metabolite activity, and time above the pharmacodynamic threshold should be linked to changes in MQC biomarkers and functional retinal outcomes.

The low bioavailability of many active NPs is largely related to first-pass metabolism in the intestine and liver after systemic administration. Different classes of compounds also show distinct absorption and metabolic profiles. Polyphenols are mainly absorbed in the upper small intestine; however, extensive intestinal and hepatic first-pass metabolism limits the systemic accumulation of their active aglycones. After absorption, these compounds are rapidly metabolized by uridine diphosphate-glucuronosyltransferases (UGTs), sulfotransferases (SULTs), and catechol-O-methyltransferase (COMT) in intestinal epithelial cells and hepatocytes into glucuronide conjugates, sulfate conjugates, or methylated products (Lu et al., 2025). These metabolites often show reduced biological activity and are more readily excreted in urine or bile. For example, resveratrol has low aqueous solubility but relatively high membrane permeability, and its oral absorption rate is therefore high, at approximately 75%. However, because of rapid intestinal and hepatic metabolism, systemic exposure remains very low, resulting in bioavailability of less than 1%. One study showed that when the dose of resveratrol was increased from 25 mg to 5,000 mg, plasma resveratrol concentrations did not increase linearly as expected (Walle, 2011). Even at the highest dose, the peak plasma concentration was only about 500 ng/mL. Other studies have shown that low-dose resveratrol reaches peak plasma concentrations within 30 min, whereas high-dose resveratrol may require 1.5–2 h to reach T_max_ (Gianchecchi and Fierabracci, 2020). Curcumin similarly exhibits low bioavailability because of poor solubility, limited gastrointestinal permeability, and rapid *in vivo* metabolism (Gutierres et al., 2015). Pharmacokinetic studies in STZ-induced diabetic rats further support this feature: oral administration of curcumin at 500 mg/kg yielded a T_max_ of 0.25 ± 0.00 h, a C_max_ of 60 ± 10 ng/mL, and a very short elimination half-life (T_1_/_2_) of 0.545 ± 0.215 h. Clinical studies likewise suggest that even very high doses, up to 12,000 mg/day, do not substantially increase circulating curcumin levels. Saponins generally show poor water solubility, which contributes directly to slow and incomplete intestinal absorption. For example, although notoginsenoside R1 is water-soluble because of its glycoside structure, its relatively high molecular weight (>500 Da) and strong polarity lead to poor intestinal mucosal permeability (Ruan et al., 2010). Some alkaloids also show oral bioavailability of less than 1%, with extremely low plasma C_max_ values. In addition, CYP450-mediated first-pass metabolism further reduces systemic exposure to active compounds, making it difficult to reach concentrations sufficient for effective DR treatment (Gong et al., 2014).

A major reason for the low bioavailability of NPs is that their physicochemical properties are often poorly matched to the requirements of absorption, distribution, metabolism, and excretion. For example, oxidative instability of polyphenols and poor water solubility of saponins reduce effective systemic exposure. In organ-specific diseases such as DR, even compounds that enter the circulation must still cross the BRB, a highly specialized physiological barrier, to reach retinal lesions. The BRB has inner and outer components (Molins et al., 2024). The inner BRB is formed by tightly connected retinal endothelial cells, pericytes, and glial cells, whereas the outer BRB consists of the retinal pigment epithelium and choroidal capillaries. This barrier exhibits strict selectivity toward exogenous substances. In addition, efflux transporters such as P-glycoprotein can actively pump incoming drugs back into the circulation, thereby reducing intraocular drug concentrations (Vellonen et al., 2018). Many NPs themselves also possess unfavorable properties for transmembrane transport, including relatively large molecular size, limited passive diffusion, and low solubility. When this restricted permeability is combined with low plasma exposure, BRB penetration becomes an even greater obstacle. Together, these factors limit the ability of such compounds to exert targeted regulatory effects on retinal mitochondrial injury.

In summary, although NPs show considerable therapeutic promise in experimental models of DR and represent valuable resources for the development of new drugs, their clinical translation is constrained by two major barriers. First, oral bioavailability is generally low, and even high-dose administration often fails to achieve therapeutically relevant systemic exposure. Second, the strict selectivity of the BRB, together with limited transmembrane transport capacity and active efflux, makes it difficult for even the small fraction of compounds entering the circulation to reach retinal lesions in sufficient amounts. These barriers are interrelated and mutually reinforcing, ultimately restricting the translation of many potentially active NPs from basic research to clinical application. Future studies should therefore prioritize strategies that directly address these bottlenecks, including structural modification to improve physicochemical properties and bioavailability, nanocarrier-based delivery to enhance BRB penetration, formulation optimization to improve chemical stability and targeting, and deeper investigation of pharmacokinetic behavior and BRB transport mechanisms across different NP classes. In addition to pharmacokinetic challenges, the clinical translation of nanoparticles has become more complicated due to the lack of standardized dosage definitions and significant differences in preparations. Dose standardization should not only focus on the quantification of a single indicator, but should cover the complete phytochemical components, because trace components jointly determine the overall biological activity of the extract. Taking curcuminoids as an example, if only the highest content of curcumin is used as a quality control indicator and demethoxycurcumin and bisdemethoxycurcumin are ignored, it is difficult to accurately reflect the complete composition and biological effects of the preparation (Obregón-Mendoza et al., 2025). In fact, in turmeric rhizome, the proportion of these three components varies depending on the geographical origin and variety: curcumin usually accounts for 75%–80%, demethoxycurcumin accounts for 15%–20%, and bisdemethoxycurcumin accounts for only 3%–5%. In addition, the overall antioxidant activity of natural extracts is not only determined by curcumin; the free radical scavenging activity of pure bisdemethoxycurcumin is weak (IC_50_ > 100 μM). However, when it coexists with curcumin and demethoxycurcumin in a C-3 mixture in a specific proportion, the mixture can closely mimic the activity level of natural extracts. Therefore, dose standardization should focus on the reproducible regulation of the complete plant chemical composition spectrum, not just the control of the content of a single marker. In practice, this can be achieved by synthesizing or standardizing methods to prepare a mixture with a clear proportion and complete composition, so as to ensure the consistency of the composition and proportion of different batches of active ingredients, and thus achieve truly reliable dose standardization in pharmacological research and even clinical applications. For insoluble nanoparticles, different preparation strategies may lead to order-of-magnitude differences in bioavailability, which highlights the necessity of unified quality control and formulation-specific pharmacokinetic characterization. A recent study related to DR directly clarified this point: free curcumin is taken up by retinal pigment epithelial cells at extremely low levels under oxidative stress and has no protective effect. In contrast, curcumin loaded in the self-nanoemulsifying drug delivery system shows stronger intracellular accumulation ability, which can induce SIRT1 expression at low concentrations (0.1–0.5 μM) and restore cell viability from 70% to more than 88%. Therefore, a natural product with low bioavailability can be converted into an effective retinal protector only through the preparation itself, without changing the active ingredients, which further emphasizes the need to conduct a specificity assessment of the preparation in DR research (Zingale et al., 2025). Such research may provide the necessary mechanism and technical basis for promoting the clinical translation of nanoparticles in DR treatment.

Future pharmacokinetic research should not be limited to reporting C_max_ and area under the concentration-time curve (AUC). The retinal pharmacokinetics/pharmacodynamic model should determine whether the drug concentration reached in the lesioned retina is sufficient to activate the expected mitochondrial target, normalize the expected MQC process, and improve the disease-related clinical endpoint parameters. Without this exposure-response relationship, the positive results of *in vitro* experiments cannot be reliably transformed into clinical applications.

### 4.2 Safety assessment of anti-diabetic retinopathy natural products: toxicity challenges

**FIGURE 6:**
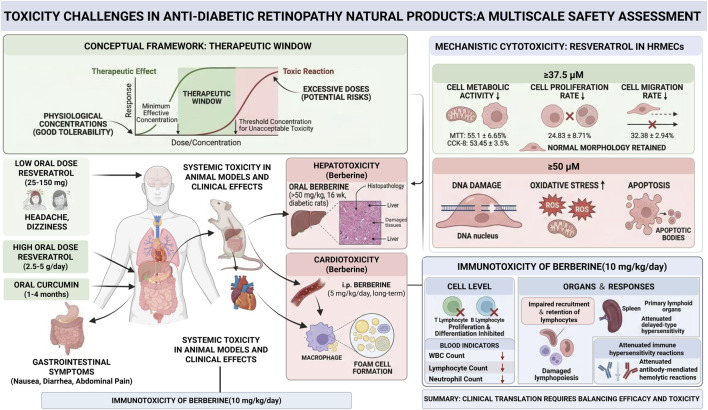
Multi-scale safety evaluation of natural products in anti-diabetic retinopathy. The diagram visualizes the toxicity challenges associated with NPs (resveratrol, berberine, curcumin) based on the therapeutic window concept. It presents dose-dependent toxic effects at three levels: 1) Cellular level (HRMEC cytotoxicity): Resveratrol at ≥37.5 µM inhibits cell metabolic activity, proliferation, and migration; at ≥50 μM, it induces DNA damage, oxidative stress, and apoptosis. 2) Systemic/clinical level: Low-dose resveratrol causes headache/dizziness; high-dose resveratrol or curcumin causes gastrointestinal symptoms (nausea, diarrhea, abdominal pain). Berberine causes hepatotoxicity (histopathology damage) at >50 mg/kg orally and cardiotoxicity (foam cell formation) at 5 mg/kg/day intraperitoneally. 3) Immunological level: Berberine (10 mg/kg/day) inhibits T/B cell proliferation/differentiation, reduces WBC, lymphocyte, and neutrophil counts, and impairs lymphocyte recruitment in the spleen. Overall, clinical translation requires balancing efficacy and toxicity.

The translational potential of therapeutic NPs depends not only on efficacy but also on safety. In this context, safety is best considered in relation to the therapeutic window, namely, the dose range between the minimum effective concentration and the threshold at which unacceptable toxicity emerges. Owing to their broad biological activities and generally favorable tolerability at physiological concentrations, NPs have attracted considerable attention in drug discovery. However, these compounds may still cause adverse effects at excessive doses or under specific conditions (Figure 6; Table 3).

**TABLE 3:** Summary of toxicological profiles of natural products intervening in diabetic retinopathy.

Classification	Natural product	Model	Route	Dose	Toxic effects	References
Polyphenols	Honokiol	Rat retinal microvascular endothelial cells	*In vitro*	50 μM	Significantly reduces cell viability, suggesting potential cytotoxicity at high concentrations	Shi et al. (2024)
​	Resveratrol	Human retinal microvascular endothelial cells	*In vitro*	37.5 μM	Significant inhibition of cell metabolism, proliferation, and migration functions (activity decreased by ∼50%), but cell morphology remains intact	Gonzalez-Perez et al. (2024)
​	Curcumin	Human foreskin	*In vitro*	40 µM	50% loss of mitochondrial activity; acute double-stranded DNA leakage at 24 h	Copetti et al. (2022)
Alkaloids	Berberine	Mouse	i.p	10 mg/kg	Reduced spleen weight and total cell count; decreased numbers of white blood cells, lymphocytes, and neutrophils in peripheral blood, suggesting immunosuppression. The absolute numbers and relative proportions of CD19^+^ B cells, CD4^+^ T cells, and CD8^+^ T cells in the spleen decreased, thereby confirming the toxic effects on immune cells	Mahmoudi et al. (2016)

Although the toxicity of many NPs is relatively low, marked cytotoxicity may occur once concentrations exceed a certain threshold. Resveratrol is generally considered safe and is widely used as a dietary supplement (Umeda et al., 2021). However, at concentrations of ≥37.5 µM, resveratrol significantly reduces cellular metabolic activity (MTT assay: 55.1% ± 6.65%; CCK-8 assay: 53.45% ± 3.5%), decreases cell proliferation to 24.83% ± 8.71%, and inhibits cell migration to 32.38% ± 2.94%, although no marked morphological changes were observed (Gonzalez-Perez et al., 2024). These findings indicate that these concentration of resveratrol markedly suppresses the metabolic activity, proliferation, and migration of human retinal microvascular endothelial cells. In addition, resveratrol at concentrations of ≥50 µM can induce DNA damage, increase oxidative stress, and promote apoptosis (Wang B. et al., 2024). Similar cytotoxic effects have also been reported for other NPs. For instance, honokiol at 50 µM significantly reduces the viability of rat retinal microvascular endothelial cells, indicating potential cytotoxicity at high concentrations (Shi et al., 2024). Likewise, curcumin at 40 µM causes approximately 50% loss of mitochondrial activity and acute double-stranded DNA leakage within 24 h in human foreskin fibroblasts (Copetti et al., 2022).

Beyond direct cytotoxicity, broader organ toxicity represents another important dimension in the safety evaluation of NPs. Resveratrol is generally well tolerated at low oral doses of 25–150 mg, with only mild adverse effects such as headache or dizziness reported in some individuals (Harley et al., 2026). However, gastrointestinal symptoms, including nausea, diarrhea, and abdominal pain, have been observed at higher doses, particularly at gram-level doses of approximately 2.5–5 g/day. Similarly, curcumin administration for 1–4 months may also cause gastrointestinal adverse reactions such as nausea and diarrhea (Shaito et al., 2020). For hepatotoxicity, oral berberine doses greater than 50 mg/kg for 16 weeks caused marked hepatic injury in diabetic rats (Zhou et al., 2008). For cardiotoxicity, long-term intraperitoneal berberine at 5 mg/kg/day promoted foam cell formation in experimental animals (Li et al., 2009).

NPs may also exhibit immunotoxicity. For example, berberine at 10 mg/kg/day significantly inhibits T-cell and B-cell proliferation and differentiation, while also reducing delayed-type hypersensitivity and antibody-mediated hemolysis (Mahmoudi et al., 2016). At this dose, berberine also markedly decreases white blood cells, lymphocytes, and neutrophil counts and impairs lymphopoiesis, lymphocyte recruitment, and splenic retention. Taken together, the translational value of NPs depends not only on therapeutic efficacy but also on an acceptable safety profile. Although many NPs are well tolerated at physiological concentrations, exceeding critical thresholds may still lead to cytotoxicity, organ injury, and immunosuppression. However, long-term safety data remain notably scarce: most preclinical toxicology studies employ only acute or sub-acute exposure, whereas DR would require years of continuous NP administration. Recent 90-day repeated-dose toxicity studies of plant-derived preparations in rodents have revealed dose-dependent hepatic and renal alterations, as well as sex-specific adverse effects, highlighting safety concerns that short-term testing cannot capture (Mathias et al., 2026). Moreover, herb–drug interactions arising from CYP450 modulation by NPs represent a significant concern for DR patients on chronic polypharmacy, yet such interactions are rarely systematically evaluated in long-term NPs safety studies (Madanahalli et al., 2026). Therefore, clinical translation requires careful balancing of efficacy and toxicity, particularly during long-term or high-dose administration.

Furthermore, a critical challenge in evaluating the true pharmacological efficacy of NPs is the potential artifacts associated with pan-assay interference compounds (PAINS). Some prominent NPs discussed in DR research, especially curcumin and resveratrol, have been described in medicinal chemistry as having PAINS-like liabilities. Because of their intrinsic chemical reactivity, these compounds may engage in nonspecific protein interactions or interfere with assay readouts in high-throughput *in vitro* systems. As a result, pronounced effects on mitochondrial markers observed at relatively high concentrations in cell culture may, in some cases, reflect assay interference or nonspecific cellular stress rather than genuine targeted remodeling of the MQC network. For rigorous clinical translation of NPs in DR, future studies should therefore incorporate orthogonal validation approaches, target-engagement assays, and robust *in vivo* pharmacokinetic-pharmacodynamic correlation analyses to better substantiate specific biological activity.

### 4.3 From barrier penetration to mitochondrial localization: nano-delivery strategies for natural products in anti-DR therapy

Due to the complex pathogenesis of DR and the strong barrier function of the BRB, traditional routes of administration often fail to achieve effective drug accumulation at retinal lesion sites, which substantially limits clinical efficacy. NPs offer potential advantages in the treatment of retinopathy because of their diverse sources, broad biological activities, and relatively low toxicity. However, their clinical translation is often limited by poor water solubility, low biological stability, rapid *in vivo* metabolism, and difficulty in crossing the BRB. With controllable particle size, good biocompatibility, and targeting capability, nanoparticle-based delivery systems may help address several of these major barriers to ocular delivery of NPs. In recent years, these systems have become an active area of research in posterior-segment ocular therapy. Among them, naturally derived exosomes and extracellular vesicles, as well as synthetic carriers such as liposomes and polymer nanoparticles, have shown considerable promise for ocular delivery of NPs. The following sections summarize representative progress in this area.

Exosomes are nanosized vesicles involved in intercellular communication and mainly transport proteins, lipids, and nucleic acids (Vukelić et al., 2026). Because of their natural structural characteristics, they can traverse biological barriers relatively efficiently, protect internal payloads, and generally induce minimal immune responses. Exosomes may therefore offer advantages over many synthetic carriers in terms of targeting accuracy and biocompatibility. Their intrinsic tropism, payload-protective capacity, low immunogenicity, and ability to cross the BRB make exosomes promising next-generation ocular drug-delivery carriers. Extracellular vesicles likewise possess nanoscale size, a lipid bilayer structure, good biocompatibility, and low immunogenicity, making them attractive drug-delivery platforms capable of crossing biological barriers, including the BRB. For example, studies using apoptotic extracellular vesicles derived from bone marrow mesenchymal stem cells showed that, after berberine loading, these vesicles maintained a particle size of approximately 200 nm and a typical cup-shaped morphology. Following intravitreal injections, they efficiently penetrated retinal tissue and were taken up by target cells (You et al., 2025). In an oxidative stress-induced retinal injury model, this delivery system reduced the cell death rate by more than 85%, significantly outperforming berberine alone. These findings support the feasibility of apoptotic extracellular vesicles as ocular-targeted drug carriers and provide evidence for the development of more biocompatible intraocular delivery systems.

In recent years, liposomes and polymeric nanoparticles have also shown clear advantages in ocular delivery studies of NPs. These carrier systems can improve NP stability, prolong circulation time, and enhance permeability across the BRB, thereby increasing drug accumulation in retinal lesions. As biomimetic vesicular structures composed of phospholipid bilayers, liposomes can encapsulate both lipophilic and hydrophilic NPs (Yue et al., 2022). They may cross the BRB through mechanisms such as membrane fusion or endocytosis, thereby reducing off-target toxicity. By contrast, polymeric nanoparticles offer controllable particle size, high drug loading, and sustained-release properties (Yang et al., 2024). They can also enable responsive drug release according to signals in the lesion microenvironment, further improving delivery efficiency. To date, various nanocarriers have been explored for natural-product delivery, with encouraging results. For example, highly branched nanocarriers, owing to their large surface area and favorable biocompatibility, can effectively encapsulate hydrophobic NPs such as curcumin and markedly improve water solubility. Surface modification can further enhance targeting capacity and delay systemic clearance, thereby helping overcome low solubility, rapid metabolism, and poor bioavailability of curcumin and providing a feasible strategy for its application in retinopathy treatment (Zhou et al., 2022). *In vitro* evaluation of curcumin-loaded solid lipid nanoparticles showed sustained release and improved stability. The results showed that this nano-delivery system extended the *in vitro* release time of curcumin to 12 h, achieving sustained release and reducing fluctuations associated with burst release of the free drug. At the same time, the carrier significantly improved the stability of curcumin in simulated body fluids and reduced degradation, thereby providing a methodological basis for subsequent *in vivo* studies. Additional animal studies in an asthma rat model showed that curcumin lipid nanocarriers increased plasma and tissue curcumin concentrations, further supporting the potential of nano-delivery systems to improve the pharmacokinetic profiles of NPs.

Importantly, this review identifies mitochondria as a core target of damage in DR. For NPs to exert therapeutic effects in DR, they must not only cross the BRB but also, more critically, reach mitochondria and penetrate the inner mitochondrial membrane to modulate the MQC network. On this basis, ocular mitochondrial-targeted drug-delivery systems are of particular interest. Such systems are increasingly being explored in this context. For example, nanocarriers modified with triphenylphosphonium and its derivatives, mitochondrial-penetrating peptides, coenzyme Q10, or oligoarginine peptides can enhance mitochondrial membrane penetration and thereby support more precise intracellular delivery of NPs. Triphenylphosphonium-modified liposomes can penetrate mitochondrial membranes through their cationic properties and deliver NPs such as curcumin and resveratrol to mitochondria in retinal pigment epithelial cells and Müller cells (Zielonka et al., 2017). This improves mitochondrial membrane potential, suppresses oxidative stress, and protects mitochondrial function. In high-glucose-induced retinal injury models, polymeric nanoparticles modified with mitochondrial-penetrating peptides showed effective targeting, significantly enhanced mitochondrial drug accumulation, improved the balance between mitochondrial fission and fusion, and reduced retinal vascular leakage and apoptosis, thus providing a potential strategy for DR treatment (Zhang et al., 2020). In addition, delivery systems such as coenzyme Q10-modified nanocarriers, oligoarginine-functionalized exosomes, and cell-penetrating peptide-coupled nanovesicles can further target mitochondria after passing through the BRB, thereby reducing nonspecific cytoplasmic retention and enhancing the regulatory effects of NPs on the mitochondrial respiratory chain, mitophagy, and mitochondrial dynamics. Some studies have shown that mitochondria-targeted liposomal delivery of NPs can reverse mitochondrial dysfunction in retinal microvascular endothelial cells under high-glucose conditions, reduce ROS levels, and improve ATP synthesis, thereby providing direct support for their potential to delay DR progression (Amato et al., 2023). These systems combine BRB penetration with mitochondrial targeting and thus support a three-step delivery paradigm of drug accumulation, cellular uptake, and mitochondrial localization, which is closely aligned with the mitochondrial-centered pathogenesis of DR.

In addition, the eMDVs-CoQ10 platform has recently shown favorable delivery efficiency and therapeutic potential in DR treatment (Gui et al., 2025). As a biologically derived nano-delivery platform, it efficiently loads coenzyme Q10 using an ultrasound-assisted approach while preserving the characteristic bilayer structure of the vesicle and stable drug-release behavior. Derived from retinal microvascular endothelial cells, eMDVs naturally retain outer and inner mitochondrial membrane proteins, including translocase of outer mitochondrial membrane 20, VDAC1, and OPA1, as well as partially active electron transport chain complexes. When administered topically as eye drops, eMDVs-CoQ10 can penetrate the corneal epithelial and stromal barriers and also cross the BRB efficiently because of its homologous properties, resulting in selective enrichment in pathological retinal microvascular regions. After cellular uptake, the eMDVs-CoQ10 platform promotes MQC-network remodeling. The platform suppresses overactivated integrated stress responses and inhibits the eIF2α-ATF4-CHOP pro-apoptotic pathway, thereby attenuating combined ER and mitochondrial stress. In diabetic rat models, long-term topical instillation markedly increased retinal ATP production, restored mitochondrial membrane potential, and significantly increased inner and outer nuclear layer thickness (INL and ONL), thus reducing photoreceptor loss (Lam et al., 2024). In addition, pathological and serological follow-up over several months suggested that this nano-eye-drop system could alleviate the progression of DR, improve anterior-segment inflammation and tear-film stability, without detectable systemic toxicity or ocular irritation. In terms of delivery efficiency, therapeutic effects, and safety, eMDVs therefore appear promising relative to conventional exosome-based platforms. More broadly, this noninvasive, penetration-enhancing, and pathway-modulating strategy may help overcome the pharmacokinetic limitations of orally administered NPs and could support future clinical translation in retinal microvascular degenerative disease.

In summary, nano-delivery systems provide a practical route for improving the clinical translatability of NPs in retinopathy by addressing key challenges such as low bioavailability, limited BRB penetration, and poor stability. Naturally derived exosomes and extracellular vesicles offer strong potential in targeted delivery and biocompatibility because of their inherent biological properties. Through structural modification and performance optimization, synthetic carriers such as liposomes, polymer nanoparticles, and highly branched nanocarriers can be adapted to the delivery requirements of different NPs and further broaden their applicability. Available studies indicate that various nano-delivery systems can enhance ocular accumulation, prolong drug action, and improve therapeutic efficacy. This is particularly relevant for mitochondria-targeted delivery strategies in DR, where such systems may help improve mitochondrial function and delay disease progression. However, some carriers still face limitations, including insufficient drug-loading capacity, uncertain long-term *in vivo* performance, and difficulties in large-scale production. Future work should therefore focus on optimizing carrier design, developing new targeting strategies, and strengthening *in vivo* safety evaluation to facilitate the clinical translation of nano-delivery systems combined with NPs and to provide more effective and safer therapeutic options for patients with DR. Furthermore, the clinical translation of nano-delivery systems for NPs requires addressing large-scale manufacturing reproducibility and the absence of harmonized regulatory frameworks for nanomedicines containing NPs, which currently limit batch-to-batch consistency and inter-study comparability (Rodríguez-Gómez et al., 2025).

However, improved delivery should not be treated as proof of mechanism. Nanocarriers may alter intracellular distribution, release kinetics, local retinal exposure, and off-target accumulation, thereby changing both efficacy and toxicity. Mitochondria-targeted systems should therefore be evaluated with quantitative retinal biodistribution, subcellular localization, payload-release kinetics, target-engagement assays, and long-term ocular safety endpoints.

## 5 Conclusion and outlook

This review redefines and elucidates DR as a progressive disease driven by the collapse of the dynamic mitochondrial quality control network, moving beyond the traditional microvascular-centered perspective. Together, mitochondrial dynamics, mitophagy, biogenesis, MAMs, and intercellular mitochondrial transfer form an integrated MQC network that supports retinal mitochondrial homeostasis. Within this framework, NPs offer a promising strategy for DR treatment because of their multitarget regulatory properties.

However, this emerging paradigm still faces substantial challenges in clinical translation and therefore requires careful evaluation. These challenges arise primarily from pharmacokinetic limitations, including low bioavailability and poor BRB penetration. Together, these factors result in insufficient delivery of bioactive compounds to retinal target tissue and thereby limit therapeutic efficacy, creating a key gap between experimental activity and clinical effectiveness. For the NPs discussed in this paper (e.g., curcumin, resveratrol, lutein), although some have already been translated into clinical use, it remains unclear whether they treat DR via the specific mechanisms proposed here. Future experimental studies should therefore place greater emphasis on clarifying this aspect to facilitate clinical translation. In addition, the safety of NPs requires careful assessment. Because NP composition is often complex and both efficacy and toxicity are dose-dependent, some compounds may exhibit cytotoxicity or organ toxicity at high concentrations. Accordingly, future studies should establish biomarker-based efficacy–toxicity systems, define therapeutic windows accurately, and standardize extraction and quality control of active ingredients. Regulation of the MQC network by NPs is also context-dependent. For example, in NPDR, enhancement of MQC activity may be required because of insufficient autophagy, while in PDR, suppression of specific components of MQC may be necessary under conditions of excessive autophagy. This stage dependence implies that different NP-based intervention strategies may be needed across stages of disease progression. To enable more precise intervention, future studies should integrate systems biology, computational pharmacology, and advanced *in vivo* imaging to dynamically define how NPs regulate the MQC network at different pathological stages of DR and thereby clarify stage-specific therapeutic windows.

At the same time, research on NPs targeting MAMs and intercellular mitochondrial transfer remains limited. Further investigation of these dimensions of network redesign may represent an important future direction. For instance, in diabetic nephropathy models, a natural product intervention was reported to activate transient receptor potential vanilloid 1, induce transient calcium influx, activate AMPK, and ultimately suppress FUNDC1 transcription (Wei et al., 2020). Because FUNDC1 is a key protein involved in MAM formation, its downregulation can reduce ER-mitochondria contact sites and limit excessive ER-to-mitochondria calcium transfer, thus alleviating mitochondrial calcium overload and dysfunction (An et al., 2024). Although this mechanism has not yet been established in DR, the shared pathological features of diabetic nephropathy and DR, including microvascular injury and oxidative stress, suggest potential translational relevance. Likewise, although no direct precedent currently exists for NPs targeting intercellular mitochondrial transfer in disease intervention, the dependence of this process on F-actin-mediated tunneling nanotubes suggests that natural macromolecules capable of modulating cytoskeletal homeostasis or improving the local microenvironment deserve further investigation. Such compounds may counteract toxic lipids that interfere with TNT formation and thereby help restore endogenous mitochondrial transfer from pericytes to microvascular endothelial cells. In this context, exploration of NP-assisted mitochondrial transplantation-based repair strategies may open new avenues for future research. To address current translational bottlenecks, promising strategies include the development of advanced delivery systems capable of crossing the BRB and the rational structural modification of NPs. The former mainly involves nano-delivery systems such as liposomes, polymeric nanoparticles, and exosomes, as well as prodrug approaches and physically assisted delivery techniques. These strategies aim to improve stability, prolong *in vivo* circulation, and enhance BRB permeability and tissue targeting, thereby increasing drug accumulation at retinal lesion sites. Structural modification, by contrast, seeks to improve solubility, metabolic stability, and membrane permeability while preserving the core pharmacophore, thus enabling the development of derivatives with improved therapeutic performance. For example, to overcome the poor stability and limited exposure *in vivo* of resveratrol, a series of derivatives have been developed using molecular hybridization and systematic modification of the ring substituents (Lu et al., 2024). Introduction of hydroxyl and amino groups, particularly in adjacent positions, was found to enhance antioxidant activity in the DPPH radical-scavenging assay, whereas methyl substitution produced only limited improvement relative to the parent compound.

In conclusion, re-examining DR through the lens of MQC network imbalance extends beyond the limitations of traditional frameworks and provides a new perspective for disease intervention. Although NPs still face major translational barriers, including pharmacokinetic constraints, safety evaluation, and context-dependent regulation, their therapeutic potential may be substantially strengthened through deeper integration of interdisciplinary approaches. Future studies should leverage emerging technologies more effectively. Single-cell and spatial omics can help define the transcriptional heterogeneity of retinal glial and endothelial cells during the progression of DR and map the spatial expression of key molecules such as VEGFA, angiopoietin 2, and IL1B, thus clarifying the spatiotemporal relationship among MQC dysregulation, inflammation, and vascular pathology and identifying specific cell targets for NP intervention (Ouyang et al., 2026). In parallel, AIDD-based integration of multi-omics data may facilitate construction of DR molecular network models, efficient identification of NPs targeting key MQC nodes, and prediction of structure-activity relationships and potential toxicities. Moreover, AI will play an indispensable role in future AI-assisted drug screening and precision medicine for DR management. Advanced deep learning and large language model integrated systems such as DeepDR-LLM and DeepDR Plus have achieved remarkable performance in multi-ethnic DR screening, accurate prediction of up to 5-year disease progression, and individualized clinical management recommendations, laying a foundation for precision-stratified prevention and treatment of DR (Dai et al., 2024; Li J. et al., 2024). In terms of drug discovery, AI high-throughput screening technology can greatly accelerate the mining and optimization of candidate NPs compounds targeting the MQC network, intelligently predict drug efficacy, organ toxicity, and optimal dose windows, and greatly shorten the preclinical and translational research cycle (Muthuraj and Chandrasekaran, 2026). From the perspective of precision medicine, AI can fuse fundus imaging features, clinical metadata, omics data and mitochondrial functional indicators to enable precise patient risk stratification, dynamically design stage-specific NPs intervention strategies, and overcome the limitations of a one-size-fits-all treatment model in traditional DR therapy. Meanwhile, explainable AI technology can interpret the pathological correlation between MQC network dysfunction and the progression of DR, guide the rational structural modification of NPs and the optimal design of nano-delivery systems, and further promote the clinical translation of NP-based therapies. Through collaborative advances in medicinal chemistry, pharmaceutics, nanotechnology, clinical medicine, omics, and artificial intelligence, it may ultimately become possible to develop therapies that support earlier intervention, protect the NVU, and delay or even reverse the progression of DR.

Overall, the most defensible translational claim is not that NPs simply “activate” or “inhibit” a single MQC pathway, but that appropriately dosed, retina-exposed, stage-matched, and cell-type-validated NPs may restore MQC network coordination. Future work should therefore prioritize human retinal validation, longitudinal models that capture metabolic memory, rigorous target engagement, orthogonal assay confirmation, and integrated long-term safety evaluation and retinal pharmacokinetic/pharmacodynamic analysis before clinical efficacy can be inferred.

## Glossary

AKT: protein kinase B
AMPK: adenosine monophosphate-activated protein kinase
AUC: area under the concentration-time curve
BAP31: B-cell receptor-associated protein 31
BNIP3: B-cell lymphoma 2-interacting protein 3
BRB: blood-retinal barrier
cGAS: cyclic GMP-AMP synthase
COMT: catechol-O-methyltransferase
DR: diabetic retinopathy
Drp1: dynamin-related protein 1
eMDVs: endosomal mitochondrial-derived vesicles
Epac1: exchange protein directly activated by cAMP 1
FIS1: mitochondrial fission 1 protein
FUNDC1: FUN14 domain containing 1
GABARAP: gamma-aminobutyric acid receptor-associated protein family proteins
GRP75: glucose-regulated protein 75
HOTAIR: HOX transcript antisense intergenic ribonucleic acid
IP3R1: inositol 1,4,5-trisphosphate receptor type 1
IP3R2: inositol 1,4,5-trisphosphate receptor type 2
LC3: microtubule-associated protein 1 light chain 3
MAMs: mitochondria-associated endoplasmic reticulum membranes
MDVs: mitochondrial-derived vesicles
MFN1: mitofusin 1
MFN2: mitofusin 2
MQC: mitochondrial quality control
mTOR: mechanistic target of rapamycin
NF-κB: nuclear factor kappa B
NPDR: non-proliferative diabetic retinopathy
NPs: natural products
NRF1: nuclear respiratory factor 1
NVU: neurovascular unit
OPA1: optic atrophy 1
PDR: proliferative diabetic retinopathy
PGC-1α: peroxisome proliferator-activated receptor gamma coactivator 1-alpha
PI3K: phosphoinositide 3-kinase
PINK1: PTEN-induced putative kinase 1
ROS: reactive oxygen species
PTPIP51: protein tyrosine phosphatase-interacting protein 51
SOD: superoxide dismutase
STING: stimulator of interferon genes
SULTs: sulfotransferases
TFAM: mitochondrial transcription factor A
TNTs: tunneling nanotubes
UGTs: uridine diphosphate-glucuronosyltransferases
VAPB: vesicle-associated membrane protein-associated protein B
VDAC1: voltage-dependent anion channel 1
VEGF: vascular endothelial growth factor.
